# Versatility of the Anterolateral Thigh Free Flap: The Four Seasons Flap

**Published:** 2012-05-03

**Authors:** Michele Di Candia, Kwok Lie, Devor Kumiponjera, Jeremy Simcock, George C. Cormack, Charles M. Malata

**Affiliations:** ^a^Department of Plastic and Reconstructive Surgery, Addenbrooke's Hospital, Cambridge University Teaching Hospitals Foundation Trust, Cambridge, UK; ^b^University of Cambridge School of Clinical Medicine, Cambridge, UK

## Abstract

**Presented at the following academic meetings:**

○ 56th Meeting of the Italian Society of Plastic, Reconstructive and Aesthetic Surgery (SICPRE) Fasano (Brindisi), Italy, September 26-29, 2007

○ 42nd Meeting of the European Society for Surgical Research (ESSR), Warsaw, Poland, May 21-24, 2008

○ Winter Meeting, British Association of Plastic, Reconstructive and Aesthetic Surgeons, (BAPRAS) London, December 1-3, 2009

**Background:** The anterolateral free flap has become increasingly popular at our institution year on year. We decided to review our experience with this flap and study the reasons for this trend. **Methods:** A retrospective review of all anterolateral thigh free flaps performed at Addenbrooke's University Hospital from the available charts was carried out. This chart review included patients' demographics, indications, flap size, recipient vessels used, ischemia time, flap, and donor site outcomes. All flap perforator vessels were located preoperatively using a handheld Doppler ultrasound probe. **Results:** From October 1999 to December 2008, 55 anterolateral thigh flaps were performed in 55 patients to reconstruct a variety of soft-tissue defects (upper and lower limbs, chest wall, skull base, head and neck). Flap size ranged 12 to 35 cm in length and 4 to 11 cm in width. During flap elevation, the main supply to the flap was found to be a direct septocutaneous perforator in 41% (n = 23) of the cases as opposed to a musculocutaneous perforator, which was found in 59% (n = 32). The mean ischemia time was 82 minutes (range, 62-103). The overall flap success rate was 100%. Two flaps were successfully salvaged after reexploration for venous congestion. The donor site morbidity was minimal. The mean follow-up time was 18 months (range, 2-48). **Discussion and Conclusion:** The anterolateral thigh free flap was found to be a very reliable flap (100% success) across a wide range of clinical indications. It facilitates microvascular anastomoses as evidenced by the short ischemia time. It provided ample skin with volume that could be tailored to the defect. These advantages have led to its widespread use by different consultants and trainees in our department.

Since its description by Song and colleagues in 1984^1^ and elucidation of its vascular anatomy by Cormack and Lamberty^2^^,^^3^ the anterolateral thigh free flap has become a popular flap in Asia,^4^^-^9 but its adoption in the West has been relatively slow.^10^^-^12

The use of the anterolateral thigh (ALT) free flap has been well described for reconstruction of defects of the head and neck,^7^^,^^8^^,^^13^^-^16 lower extremities,^17^^-^19 upper limbs,^20^^-^22 breast,^23^^,^^24^ and trunk.^7^^,^^25^^,^^26^ The large skin island, easy microsurgery, reliability, and versatility (skin, fat, muscle) have combined to make it one of the preferred flap choices for soft-tissue defect reconstruction.^27^^-^29

It is a reliable flap that supplies a large area of skin, it can be harvested as either a septum-cutaneous or a muscle-cutaneous flap, and the thickness and volume can be adjusted to match the defect. Therefore, it can replace most soft-tissue free flaps in most clinical situations.

There are few papers, which have evaluated its overall usefulness or versatility for a variety of different tissue defects and none of these have been in Western populations.^27^^-^30 Since the arrival of new consultants at our institution, it was noted that the applications of the ALT in our unit had widened.

We thereby decided to review our 16-year experience with 49 ALT flaps used for a variety of soft-tissue defects and highlight the reasons for its versatility and benefits.

## PATIENTS AND METHODS

A retrospective study of all patients undergoing free ALT flap reconstruction at Addenbrooke's University Hospital between October 1992 to December 2008 was carried out. Patients were identified by a manual search of the operating theater records and cross-referenced with the consultants' logbooks. Case notes were searched for demographic information, pathologic diagnosis, defect location, flap size, flap anatomy, technique of flap harvest, receipt vessels used, ischemia time, flap outcome, and donor site complications.

### Flap planning and elevation

The ALT flap was raised with the patient in supine position as described by Cormack and Lamberty.^31^ A line is drawn between the anterior superior iliac spine and the superolateral border of the patella. This determines the axial line of the flap. The location of the main cutaneous perforators from the descending branch of the lateral circumflex femoral artery (LFCA) was detected with a Doppler ultrasound probe using an 8-MHz transducer and marked on the skin. The majority of these vessels are located in the inferolateral quadrant of a circle, with a radius of 3 cm if drawn from the midpoint as center. The flap is designed over the location of these vessels with its long axis parallel to that of the thigh. The flap may be reliably designed with the perforating vessels at the superior end of the skin flap to maximize pedicle length if required. Nine flaps were raised by using a sterile Esmarch tourniquet secured to a Steinman pin in the anterior superior iliac spine in the manner previously described by Cormack in 1992.^32^^,^^33^

Flap elevation starts at the medial edge and the skin is incised including the deep fascia of the thigh. The tissues are elevated until the intramuscular septum between rectus femoris and vastus lateralis is visualized. The lateral cutaneous nerve of the thigh will be encountered in the subcutaneous fat in its distal course at the anterosuperior edge of the flap. If a sensate (neurosensory) flap is desired, it may be included in the elevation.^34^ Motor branches of the femoral nerve to the lowermost part of vastus lateralis may be encountered deeply in the septum and should be preserved as much as possible.

If the perforator is septum-cutaneous, the vessels to the skin can be seen at this level, the dissection is straightforward and uncomplicated until the main pedicle is reached. In the absence of a septum-cutaneous perforator, it is necessary to elevate the flap until the perforator is identified emerging from the muscle and then to trace it back through the anterior part of vastus lateralis to its origin from the descending branch of the lateral femoral circumflex artery.

The dissection of the pedicle usually terminates at the point where the arterial branch to rectus femoris arises and its venous drainage joins unless a longer pedicle is required. The donor site is usually closed directly. If it is not possible to close the defect directly because a large skin paddle has been harvested, then the skin edges can be advanced and sutured to the muscle to reduce the size of the defect and the remaining area can be closed with a skin graft taken from the medial aspect of the same thigh.

## RESULTS

From October 1999 to December 2008, 55 free ALT flaps were transferred to reconstruct soft-tissue defects of the extremities, trunk or head and neck in 55 patients. There were 39 male and 16 female with a mean age of 52 years (range, 13-73). Twenty-one ALT flaps were used for upper and lower extremities, 13 for skull base, 15 for head and neck region, and 6 for the trunk (Table 1).

In 21 patients, the ALT was performed following trauma and in 34 to repair defects resulting from excision of malignant tumors. Numerous recipient vessels were used. The flap size ranged from 12 to 35 cm in length and 3 to 11 cm in width. All flap perforator vessels were located preoperatively using a handheld Doppler ultrasound probe and their position confirmed at the time of flap harvest. In no cases were the detected perforator vessels either absent or arising from a vessel other than the lateral femoral circumflex artery (LCFA). The duration of surgery from induction of general anesthesia to leaving the operating room averaged 9.3 hours (range, 5.5-11.5). None of the patients had any intraoperative complications. The mean flap ischemia time was 86 minutes (range, 62-103) (Table 2).

During flap elevation, a direct septum-cutaneous perforator was observed in 11 patients (22%); in all these cases, the ALT was transferred as a fasciocutaneous flap. In 38 patients (78%), a muscle-cutaneous perforator was found. Of the muscle-cutaneous perforators, intramuscular dissection of a true perforator flap was carried out in 14 cases (37%) and a cuff of vastus lateralis muscle was taken with the perforator in 24 patients (63%) either for safe elevation of the flap, to fill deep defect and/or to cover irregular exposed bone. In 3 patients, the lateral femoral cutaneous nerve was also included for a sensate flap respectively for a total tongue and 2 hand reconstructions. In 4 cases (3 hands and 1 forehead reconstruction), the ALT flap was thinned leaving a 5 to 6 mm layer of subcutaneous fat.

The donor site was closed directly in 42 patients (79%) and a meshed split skin graft was used in 13 (21%). Two flaps required reexploration of the microvascular anastomoses for venous congestion and both were successfully salvaged.

In 6 patients (12%), donor site problems were observed. These included 2 cases of partial skin graft taken, 3 persistent seromas that required few aspirations to resolve, and 1 hematoma evacuated in theater. The muscle power of the quadriceps of the donor thigh after harvesting ALT muscle-cutaneous flap did not obviously affect the strength or stability of the donor knee and therefore no donor site problems were noted in the long follow-up.

All flaps remained healthy during an average follow-up of 18 months (range, 5-38) (Table 3); all patients had good volume filling of their defects and were generally satisfied with the outcome.

## DISCUSSION

The ALT flap is based on the septum-cutaneous and/or muscle-cutaneous perforators from the descending branch of the LCFA. This vessel sends perforators through the septum between the vastus lateralis and the rectus femoris muscles or through the substance of vastus lateralis, supplying a large skin paddle on the anterolateral aspect of the thigh. If a septum-cutaneous perforator is found, the flap can be harvested as a septum-cutaneous flap,^34^ which is the ideal for most indications having the lowest morbidity in terms of donor site. However, if septum-cutaneous perforators are absent, the flap can then be harvested as a muscle-cutaneous flap, with varying amount of vastus lateralis muscle for added bulk, or as a true perforator flap following intramuscular dissection of the perforators.^35^^,^^36^

Careful preoperative planning in flap design is essential. Although different techniques have been recommended for the preoperative assessment and localization of the cutaneous perforators,^32^^,^^35^^,^^37^ the Doppler audiometer was found to be perfectly adequate in all our cases, as confirmed during flap harvest. It is, however, necessary to be familiar with the anatomical variations during flap elevation^32^ because most uncommonly, 2 descending branches of the LCFA run in parallel in the septum,^2^^,^^3^^,^^6^^,^^23^; therefore, it is particularly important to identify the deeper of these before dissecting out the pedicle.

**Figure 1:**
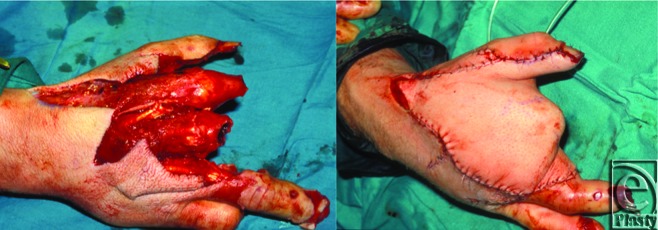
A 32-year-old man who sustained blast trauma to his right hand with amputation of the index and middle fingers at the metacarpophangeal joint level. A fasciocutaneous ALT free flap anastomosed to the radial artery and cephalic vein was properly tailored to cover the exposed metacarpal bones and repair the soft tissue defect.

**Figure 2:**
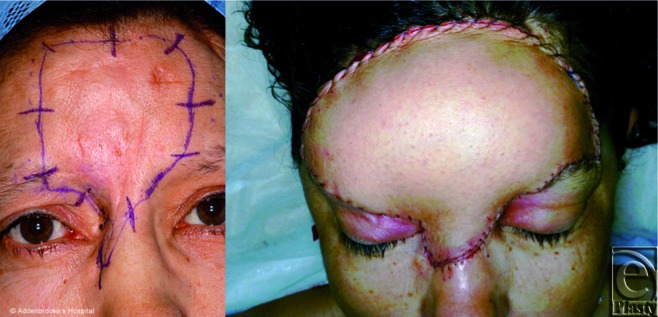
A 49-year-old lady plagued by repeated recurrence of a dermatofibrosarcoma protuberans of the forehead underwent extensive resection that required a complete removal of both frontalis muscles. She was reconstructed with a thinned muscle-cutaneous ALT free flap anastomosed to superficial temporal vessels. It was vital to orientate the flap appropriately with respect to the pedicle location.

Compared with other free flaps, the ALT flap has numerous advantages (Table 4), including the following: (1) the skin paddle is pliable and can be easily trimmed for any area that has to be reconstructed^8^^,^^9^^,^^11^^,^^13^ (Fig 1); (2) a large skin area that can be harvested even when only a single major cutaneous perforator is available^30^^,^^36^^,^^37^; (3) the flap anatomy allows safe modifications of its volume as required: in our series, we thinned successfully 4 flaps (3 hands, 1 forehead) (Fig 2) down to a 5 to 6 mm uniform layer of fat under loupe magnification except within a 2 cm radius of the vascular pedicle, carefully preserving the small veins beneath the dermis, as previously described.^11^^,^^15^^,^^22^ We did not encounter any complications of thinning such as skin necrosis, inadvertent perforator, or pedicle division or bleeding from the under-surface of the flap; (4) a long vascular pedicle can be obtained, up to 16 cm.^27^^,^^28^^,^^29^^,^^30^^,^^35^ This allows a wider choice of recipient vessels as illustrated in our series; (5) it can be a sensate flap by including the anterolateral femoral cutaneous nerve—this is mandatory in hand reconstruction and advantageous in tongue reconstruction, even though its dissection takes slightly longer 11^,^^20^^,^^22^^,^^34^; (6) there is minimal morbidity at the donor site, the functional deficit following flap elevation was minimal and temporary even when a portion of vastus lateralis muscle was included. The vastus lateralis muscle is a syn-agonist of the other 3 knee extensors and its removal does not affect leg function. Occasionally, when the motor nerve to the lower part of the vastus lateralis passes between 2 perforators that are going to the flap, it requires to be divided to free the flap, then the nerve is divided as far distally as possible and repaired with the usual microsurgical techniques.

**Figure 3:**
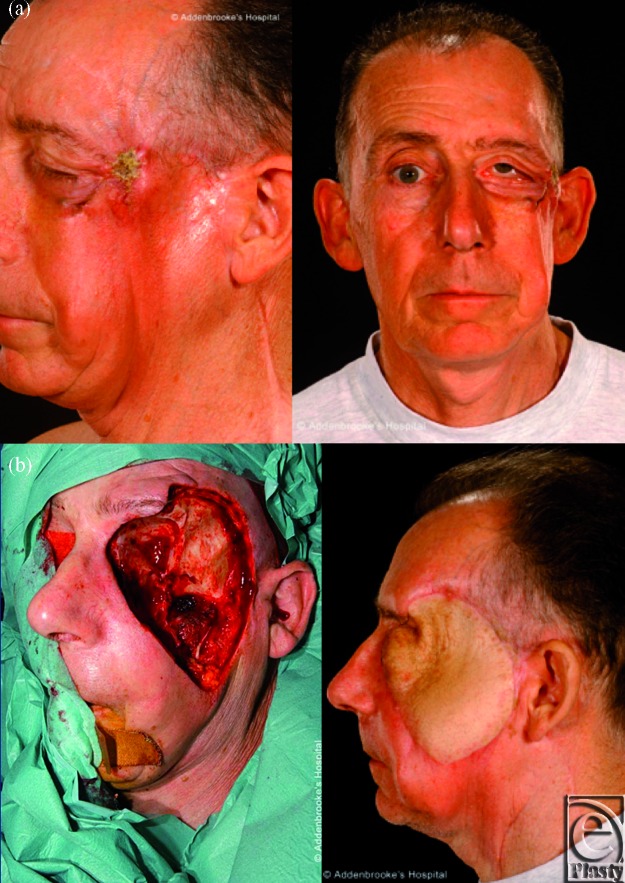
A 65-year-old man affected by recurrence of invasive basal cell carcinoma of the left orbital rim and left zygoma, previously treated with surgery and radiotherapy (Fig 3a) underwent wider resection, partial maxillectomy and exenteration of the left ocular globe. The resultant defect was repaired using a muscle-cutaneous ALT free flap anastomosed to the facial artery and internal jugular vein. It allowed a reasonable functional and aesthetic outcome (Fig 3b)

**Figure 4:**
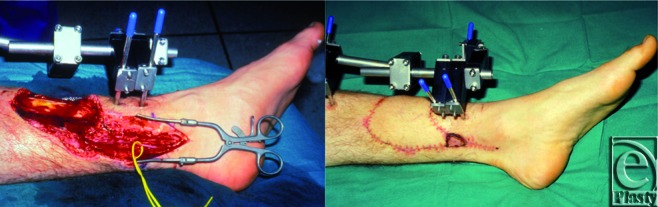
A 29-year-old man with an open posttraumatic fracture of the right tibia. A muscle-cutaneous ALT free flap with a small cuff of vastus lateralis, anastomosed to the posterior tibial artery and long saphenous vein, was used to cover the bone and repair the soft tissue defect.

**Figure 5:**
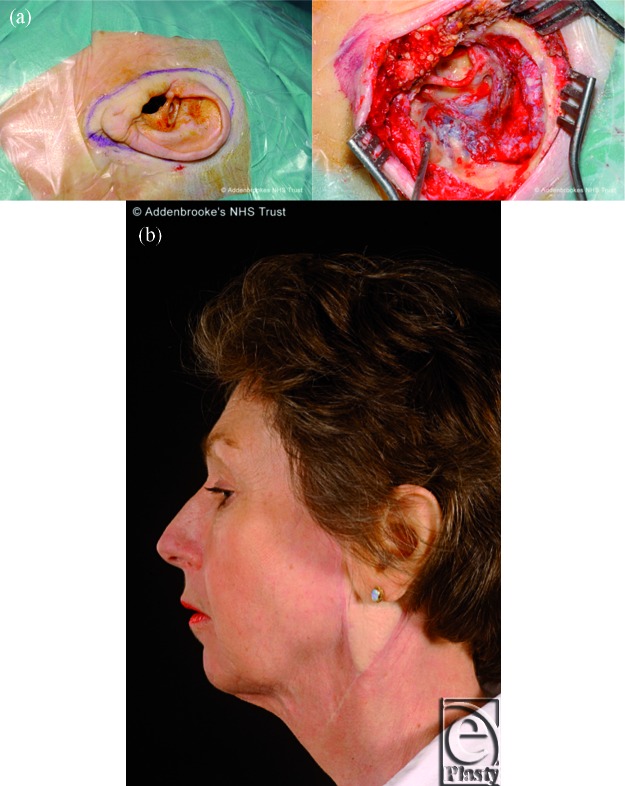
A 57-year-old lady undergoing wide resection of the left ear and of the temporal bone down to the dura mater (Fig 5a) for osteo-radio-necrosis following previous treatment for a squamous cell carcinoma of the external auditory meatus. The resulting defect was reconstructed using a fascia latae graft and a muscle-cutaneous ALT free flap with a small cuff of vastus lateralis anastomosed to the lingual artery and the internal jugular vein. The outcome was acceptable after 18 month follow-up. Please note the poor color match of the flap in relation to the recipient site (Fig 5b).

**Figure 6:**
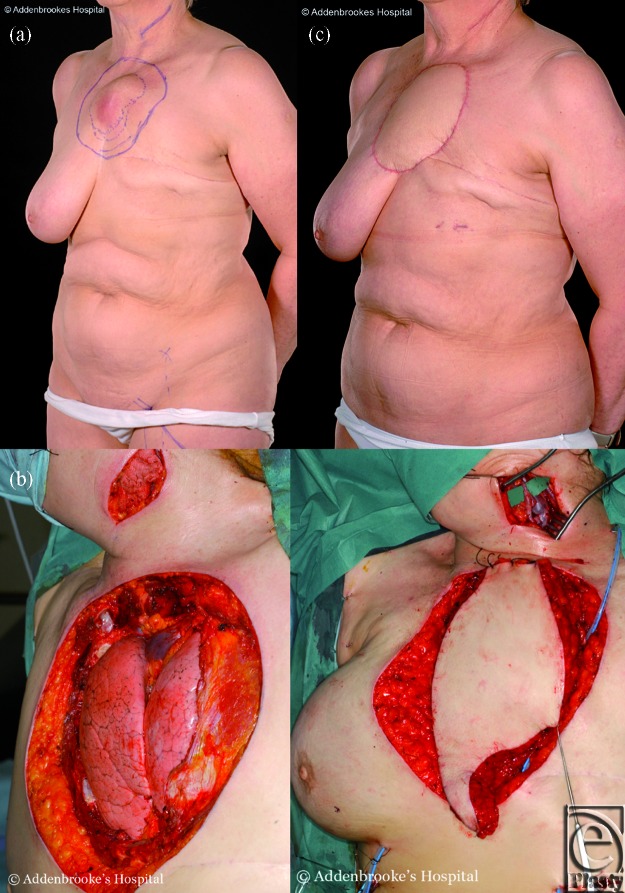
A 64-year-old lady with a large mass over the sternum caused by recurrence of previous left breast cancer (Fig 6a), who required partial resection of the sternum and the adjacent 4 pairs of ribs. Skeletal stabilization was achieved using composite synthetic materials. A muscle-cutaneous ALT free flap with a small segment of the vastus lateralis muscle was anastomosed to the facial artery and internal jugular vein to repair the soft tissue defect (Fig 6b) allowing a good functional and aesthetic result confirmed at 14 months follow-up (Fig 6c).

We grafted the donor site in 27% of cases but the skin graft did not influence the sliding of the vastus lateralis muscle. There was no change in gait at postoperative follow-up^23^^,^^37^^,^^38^; (7) It is easily harvested in the supine position, which is the safer and more comfortable position for the patient.^23^^,^^29^^,^^30^ (8) The location is usually distant from most defects allowing comfortable 2-team operating. (9) Thanks to its vascular anatomy that can be anastomosed as a flow-through pedicle, a situation which is very helpful in reconstructions of extremities avoiding the use of vein grafts. (10) In addition, by incorporating part of the vastus lateralis muscle in the flap, the ALT combines the benefits of a skin flap superficially with the benefits of muscle deeply (Figs 3a-3b). The muscle component is particularly useful for covering exposed fractures (Fig 4), osteomyelitis or for filling irregular defects such as following lateral temporal bone resection^9^^,^^10^^,^^12^^,^^13^ (Figs 5a-5c), or chest wall resection when the sternum is removed^25^^,^^26^ (Figs 6a-6b).

(11) No less important is that it is an easy flap to raise and anastomose (large caliber vessels), hence the operating time is not overly long; in our series, a lot of time was spent on tumor resection and for anesthetic preparation.^33^^,^^35^

**Figure 7:**
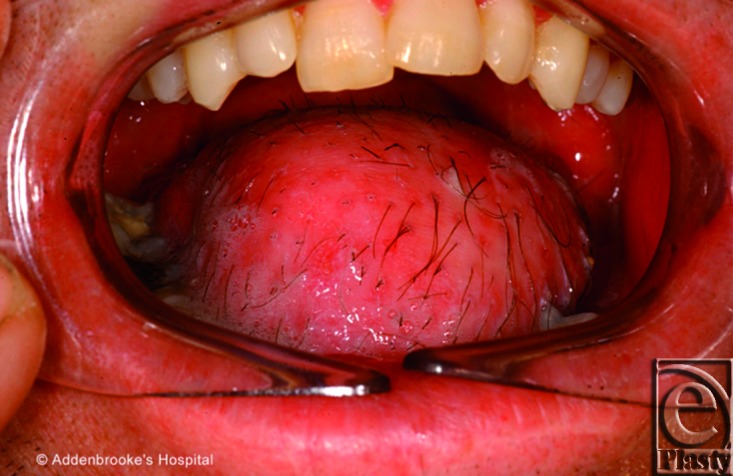
This 48-year-old man had a partial glossectomy for a squamous cell carcinoma of the tongue reconstructed with a thinned fascio-cutaneous ALT free flap. Although the postoperative outcome was acceptable in terms of bulk and shape and function, the presence of the hairy skin represented a discomfort for the patient.

There are some disadvantages to this flap. The donor site scarring associated with use of skin grafts in a large defect may not be acceptable, particularly in female patients. The high incidence of hairy skin in this area in men, especially in Caucasians may also not be desirable in particular for oral defect reconstruction (Fig 7). This is not a problem for the majority of head and neck cancer cases, as the majority subsequently have postoperative radiotherapy. We also believe that the ALT flap is not appropriate in obese patients, particularly women in which often a skin graft is required to repair the donor site (Table 5).

Variation in the origin and course of the supplying perforators are a theoretical problem largely described^35^^-^37; however, we did not find that this was a cause for abandoning flap elevation in any cases in our series.

Over the 16-year period, there has been a diversification in the indications for the ALT free flap in our department. Initially most of the ALT flaps were performed principally for extremity surgery reflecting the specialist interest of the existing consultant (G.C.C.) but with the enlargement of the plastic surgery service (2 new consultants C.M.M. and J.W.S.) and the increasing number of its publications in the literature, the use of the ALT free flap has been extended mainly outside the limbs, therefore routinely adopted for head and neck, intraoral, skull base, and chest wall reconstruction.

The donor site should be convenient for harvest, remain inconspicuous, and cause low morbidity. The ALT flap fulfils many of these requirements, and it is adaptable to a wide range of clinical situations.

## CONCLUSION

In summary, in our experience, the anatomical and logistical features of the ALT free flap make it the flap of choice in a wide range of soft tissue reconstructive situations. It contains the right tissue components in the correct amounts to fulfill the functional and cosmetic requirements of the recipient defect; it is harvested from a convenient location, in a convenient position with minimal donor site morbidity. Flap design only requires a handheld Doppler and a measuring tape. The flap, thanks to its easy orientation, can be tailored to the individual's defect by positioning for pedicle length, incorporating skin/muscle/sensory nerve and thinning for a perfect contour.

For all these advantages, the number of ALT free flaps has increased in our Department in the last few years. In addition the learning curve for this flap is faster than other perforator flaps, allowing trainees to improve their confidence with the free tissue transfer. We feel that it can represent a valid substitute for most of the commonly used soft-tissue flaps.^38^

## Figures and Tables

**Table 1:** Patients demographics (n = 55 patients)

	Number	Frequency
Male	39	71%
Female	16	29%
Age	52 (range, 13–73)	
Flaps harvested	55	
Following tumors excision	34	62%
Following trauma	21	38%
Defect site		
Upper limb	9	16%
Lower limb	12	22%
Skull base	13	24%
Trunk	6	11%
Head and neck	15	27%

**Table 2:** Overall operative findings in 55 ALT free flaps harvested in 55 patients

	Number/Average	Frequency
Flap anatomy and type		
Musculocutaneous	32	59%
True perforator flap	14	
Thinned	1	
With a cuff of muscle	18	
Sensate	2	
Septo/fasciocutaneous	23	41%
Thinned	3	
Sensate	1	
Flap size (average)		
Length, cm	24 (range, 12–35)	
Width, cm	9 (range, 4–11)	
Operative time, h	9 (range, 5 -11)	
Ischemia time, min	86 (range, 62–103)	
Length of pedicle, cm	12 (range, 10–16)	
Donor site closure		
Direct closure	42	79%
Skin graft	13	21%

**Table 3:** Outcomes in 55 reconstructions using the ALT free flaps

	Number	Frequency
Flap survival	55	100%
Total lost	0	
Partial flap necroses	0	
Flap complications		
Arterial	0	
Venous congestion	2	4%
Reexploration	2	4%
Hematoma	0	
Donor site complications		
Infection	0	
Hematoma	1	2%
Seroma	3	5%
Skin graft lost	0	
Partial skin graft lost	2	4%
Average follow-up, mo	18 (range, 2–48)	

**Table 4:** Advantages of the ALT free flap in soft tissue reconstruction

Skin paddle (large, pliable, reliable even on single perforator)
Long vascular pedicle
Large vessels enabling easy microvascular anastomoses
Favorable vascular anatomy allows thinning of the flap
Distant from many operative sites
Harvest in supine position
Can be made sensate
Inclusion of the fascia lata
Variable bulk
Low donor site morbidity
Easy flap to learn

**Table 5:** Disadvantages of the ALT free flap in soft tissue reconstruction

Variable vascular anatomy
Poor color match in head and neck reconstruction
Not suitable for obese population
High incidence of hairy skin in Caucasian
